# Cardiovascular Disease Risk Assessment from Retinal Fundus Images Using a CSP-ConvNext Model

**DOI:** 10.3390/diagnostics16183058

**Published:** 2026-09-20

**Authors:** Dhayanithi Jaganathan, Sathiyabhama Balasubramaniam, Harini Soundharyaa T, Seshathiri Dhanasekaran

**Affiliations:** 1 Department of Computer Science and Engineering, Sona College of Technology, Salem 636005, India; sathiyabhama@sonatech.ac.in (S.B.); harinisoundharyaa.24mecse@sonatech.ac.in (H.S.T.); 2 Department of Computer Science, UiT The Arctic University of Norway, 9037 Tromsø, Norway

**Keywords:** medical imaging, retinal fundus image analysis, cardiovascular disease prediction, deep learning

## Abstract

**Background/Objectives:** Cardiovascular diseases (CVDs) remain the leading cause of mortality worldwide, making early identification of individuals at elevated cardiovascular risk essential for preventing major adverse cardiac events (MACE). Retinal fundus photographs provide a non-invasive window into systemic vascular health, as retinal microvascular and optic-disc characteristics have been associated with cardiovascular abnormalities. This study aims to develop an enhanced ConvNeXt-based deep learning framework, termed CSP-ConvNeXt, for automated prediction of CVD-related risk categories from retinal fundus images, including Mild, Moderate, Severe, and Proliferative Diabetic Retinopathy (DR), as well as Hypertensive Retinopathy (HR). **Methods:** The proposed CSP-ConvNeXt framework integrates a hierarchical convolutional backbone with Cross Stage Partial (CSP) channel partitioning and large-kernel depthwise convolutions to capture both fine-grained retinal microvascular features and broader anatomical structures associated with cardiovascular risk. An Efficient Squeeze-and-Excitation (ESE) attention mechanism is incorporated to enhance the representation of clinically relevant vascular regions while reducing the influence of irrelevant background information. A robust data-augmentation and preprocessing pipeline is employed to improve model generalizability under varying retinal imaging conditions. Two benchmark retinal datasets, comprising Mendeley Retinal Fundus images and the APTOS 2019 dataset, are used for model training and evaluation. **Results:** The CSP-ConvNeXt framework achieves an overall classification accuracy of 87%, demonstrating effective discrimination among the considered CVD-related retinal risk categories. The model achieves high precision and recall values, indicating consistent classification performance across the evaluated categories. Statistical analysis demonstrates significant interclass differentiation, with a *p*-value < 1 × 10^−16^, supporting the discriminative capability of the learned retinal representations. **Conclusions:** The results demonstrate that the CSP-ConvNeXt framework, combining CSP-based feature extraction, ESE attention, large-kernel depthwise convolutions, and hierarchical representation learning, can effectively capture retinal vascular and anatomical patterns associated with cardiovascular risk. The results highlight the potential of advanced ConvNeXt-based architectures for automated, non-invasive CVD-related risk screening using retinal fundus photographs. This approach may provide a valuable complementary tool for early cardiovascular risk assessment, particularly in settings where conventional cardiovascular screening is less accessible.

## 1. Introduction

### 1.1. Cardiovascular Disease (CVD) Diagnosis and Clinical Importance

Cardiovascular disease (CVD) remains the leading cause of mortality worldwide, accounting for nearly 17.9 million deaths annually across all age groups. CVD encompasses a broad spectrum of disorders, including coronary artery disease, vascular disease, congenital heart disease, heart rhythm disorders, and heart failure [1]. More than 80% of CVD-related deaths are attributed to heart attacks and strokes, and almost one-third of these deaths occur prematurely in individuals younger than 70 years of age [2].

Unhealthy lifestyles, including poor diet, physical inactivity, smoking, alcohol consumption, obesity, hypertension, diabetes, and dyslipidemia, significantly contribute to the development of CVD. Early identification of high-risk individuals is therefore essential for reducing mortality and improving clinical outcomes. However, conventional cardiovascular risk assessment methods often rely on invasive procedures, biochemical investigations, and specialized diagnostic infrastructure, limiting their large-scale implementation in resource-constrained healthcare settings [1,2].

Retinal fundus imaging has emerged as a promising, non-invasive, low-cost, and widely accessible alternative for cardiovascular risk assessment. The retinal microvasculature shares anatomical and physiological similarities with the coronary and cerebral circulatory systems. Changes in retinal vessels often reflect systemic vascular abnormalities associated with hypertension, diabetes, and cardiovascular disorders. Consequently, retinal imaging provides a unique opportunity for the early prediction and monitoring of cardiovascular health [3].

### 1.2. Deep Learning and Convolutional Neural Network (CNN)-Based Models Using Retinal Fundus Images

The rapid advancement of Artificial Intelligence (AI), Machine Learning (ML), and Deep Learning (DL) has significantly transformed disease diagnosis and management. Unlike traditional ML methods that depend on handcrafted feature extraction, DL models automatically learn hierarchical and discriminative representations directly from raw data, resulting in improved predictive performance [4,5].

In recent years, retinal fundus imaging combined with DL techniques has gained considerable attention as an auxiliary tool for cardiovascular risk prediction [3]. DL-based Computer-Aided Diagnosis (CAD) systems [4,5,6,7] have demonstrated human-level performance in predicting several cardiovascular risk factors, including age, gender, smoking status, hypertension, diabetes, blood pressure, and Major Adverse Cardiac Events (MACE) [3] from retinal images [4,5].

Several studies have investigated the relationship between retinal biomarkers and cardiovascular disease. A systematic review identified 26 studies that predicted 42 CVD-related outcomes from retinal images, including cardiovascular risk factors, cardiac imaging biomarkers, risk scores, and incident CVD events [5,6]. Similarly, retinal vessel caliber has been recognized as an important predictor of coronary heart disease risk [7].

The emerging field of oculomics further strengthens the role of retinal imaging in systemic disease prediction by analyzing retinal vascular morphology, vessel caliber, tortuosity, curvature, and branching patterns [8,9]. Studies based on retinal image datasets such as Diabetic Retinopathy Database (DIARETDB), Structured Analysis of the Retina (STARE), Methods to Evaluate Segmentation and Indexing Techniques in the Field of Retinal Ophthalmology (MESSIDOR), and E-ophtha have demonstrated significant associations between retinal biomarkers and systemic diseases [9,10].

Recent CAD systems have also incorporated multimodal data, including retinal images, electronic health records, genomics, wearable sensor data, and clinical information, to improve disease prediction and personalized treatment planning [11]. AI-assisted diagnostic systems reduce diagnostic time, support large-scale screening programs, and provide objective assessments that minimize inter-observer variability [12,13,14]. In addition to detection, CAD models can support disease staging, disease progression, and disease outcome prediction, which are essential for the individual to plan treatment and monitor therapy. A study [15] that used Gradient Class Activation Map (GradCAM) to highlight the areas of interest in the retinal images that influenced the decisions of the DL model. The model focused mostly on the center of the retinal images, where there were signs of CVD such as hemorrhages, and used certain prognosis markers to identify hypertension and ischemic heart disease [15]. Most of these works have utilized Convolutional Neural Networks (CNNs) to process these retinal fundus images; the models were augmented with traditional risk factors and the results are prospective, which can strengthen further research in the field.

### 1.3. Limitations of Existing Methods

Despite significant progress, several limitations still prevent the widespread clinical adoption of retinal image-based CVD prediction models. Most of these approaches primarily employ CNN architectures that focus on local image features but fail to capture long-range spatial dependencies within retinal images. Although CNNs effectively extract fine-grained vascular features, they often struggle to model global retinal structures, including the relationship between the optic disk, macula, and the overall retinal geometry.

Current models also exhibit challenges, which include the following:

- Inadequate representation of both local vascular structures and global retinal features.
- Limited incorporation of anatomical priors, such as vessel saliency and optic disk information.
- Reduced generalizability across diverse datasets and imaging environments. Suboptimal performance in multi-class cardiovascular risk prediction.
- Clinical applicability remains limited because of concerns regarding robustness, interpretability, scalability, and external validation [3,15].
- Integration of retinal image analysis into routine clinical practice still requires prospective clinical studies to address ethical, regulatory, and medico-legal concerns [16,17].

### 1.4. Need for Cross-Stage Partial ConvNeXt (CSP-ConvNeXt) Architecture

Recent studies have shown that hybrid architectures combining CNNs and Vision Transformers (ViTs) can effectively integrate local feature extraction with long-range dependency modeling, leading to improved performance in medical image analysis [18]. However, many existing hybrid models do not explicitly incorporate anatomy-aware learning mechanisms. Since cardiovascular risk prediction from retinal images depends heavily on vessel morphology, optic disk characteristics, and global retinal organization, there is a need for a more robust and clinically interpretable framework.

To address these challenges, this research proposes a Cross-Stage Partial ConvNeXt (CSP-ConvNeXt) architecture that combines the advantages of CSP connections, ConvNeXt convolutional blocks, and anatomy-aware attention mechanisms. The proposed framework is designed to simultaneously capture the following:

- Fine-grained vascular features.
- High-resolution retinal textures.
- Optic disk information.
- Global retinal spatial relationships.

The incorporation of enhanced Squeeze-and-Excitation (ESE) attention and multi-source retinal datasets aims to improve model robustness, generalizability, and clinical reliability in non-invasive cardiovascular screening [19].

### 1.5. Research Contributions

The primary objective of this research is to develop and evaluate a CSP-ConvNeXt-based DL framework for predicting cardiovascular risk factors and associated diseases using retinal fundus images. The major contributions of this work are as follows:

We developed a CSP-ConvNeXt architecture capable of simultaneously learning local retinal vascular features, including microvessels, tortuosity, and hemorrhages, together with global retinal structures such as the optic disk and macula [20]. Integration of anatomy-aware learning through enhanced Squeeze-and-Excitation attention and hierarchical feature extraction improved the identification of CVD-associated retinal biomarkers [19].

We developed a robust multi-class classification framework using large-scale retinal datasets such as Mendely and Asia Pacific Tele-Ophthalmology Society (APTOS), together with extensive preprocessing and data augmentation strategies to improve model generalizability. Comprehensive evaluation of the proposed model used clinical performance metrics, including accuracy, precision, recall, F1-score, and statistical validation. Comparative analysis with existing DL approaches demonstrated the effectiveness of the proposed framework for non-invasive cardiovascular screening.

This study hypothesizes that the proposed CSP-ConvNeXt framework, combined with vessel-aware learning and anatomy-guided feature extraction, can significantly improve the prediction of cardiovascular risk factors and major adverse cardiovascular events (MACE) directly from retinal fundus images, thereby enabling scalable, affordable, and early population-level cardiovascular screening [21,22].

## 2. Literature Review

### 2.1. Traditional CAD Systems for Retinal Fundus Image Analysis

The retina provides a unique, non-invasive window into the human microvascular system. Changes in retinal vasculature, including vessel caliber, tortuosity, branching patterns, microaneurysms, and optic disk abnormalities, are strongly associated with both current cardiovascular status and long-term cardiometabolic risk. Consequently, retinal fundus imaging has emerged as a promising biomarker for CVD screening and risk stratification [3].

Traditional CAD systems primarily relied on hand-crafted feature extraction techniques combined with conventional ML algorithms. These systems used manually engineered features such as vessel width, vessel tortuosity, fractal dimensions, arteriovenous ratios, and optic disk characteristics to predict cardiovascular abnormalities. Although these approaches provided clinically interpretable results, they required extensive domain expertise and were highly dependent on feature engineering. Furthermore, their performance was often limited by variations in image quality, illumination, acquisition devices, and patient demographics.

The evolution of AI has gradually shifted retinal image analysis from handcrafted feature-based approaches toward automated representation learning using DL models. This transition has significantly improved the ability to extract complex, high-dimensional patterns that are difficult to identify through conventional image processing techniques [23].

### 2.2. CNN- and Deep Learning-Based Retinal Fundus Image Classification Models

The introduction of CNNs transformed retinal image analysis by enabling end-to-end learning directly from fundus photographs. CNN-based models automatically learn hierarchical features from retinal images, thereby eliminating the need for manual feature extraction. Recent progress in DL has substantially enhanced the prediction of conventional cardiovascular risk indicators, including age, blood pressure, smoking status, and MACE [3,24].

Several studies have demonstrated the effectiveness of DL for retinal fundus image classification, disease diagnosis, and cardiovascular risk prediction [24,25,26]. However, many of these studies have been conducted using convenience datasets with limited ethnic and geographical diversity, raising concerns regarding model generalizability and algorithmic bias. Table 1 summarizes representative CNN-based retinal fundus image classification models.

Despite these advancements, CNN-based models often struggle to capture long-range spatial relationships across retinal structures because of their localized receptive fields. This limitation has encouraged researchers to investigate transformer-based architectures.

### 2.3. Vision Transformer (ViT) Models for Retinal Fundus Image Analysis

ViTs have recently gained considerable attention in medical image analysis because of their ability to model long-range dependencies through self-attention mechanisms. Unlike CNNs, which process information using convolutional filters, Transformers divide an image into patches and analyze global relationships among these patches [30].

The rapid development of DL-based retinal image analysis has identified several important challenges, including dataset bias, ethnic variability, model calibration, explainability, and clinical validation. These studies have emphasized the need for anatomically informed and multimodal architectures that can improve the reliability of cardiovascular risk prediction systems [30,31,32].

ViT models have demonstrated better performance in several medical imaging applications, including image classification, segmentation, and prognosis prediction. Their ability to capture global contextual information makes them particularly suitable for retinal image analysis, where disease-related biomarkers may be distributed across multiple retinal regions. However, transformer models typically require very large training datasets and substantial computational resources. In addition, their clinical interpretability remains an important challenge.

### 2.4. Hybrid CNN–Transformer Models

Recent studies have shown that combining CNNs and transformers can effectively integrate the strengths of both architectures. CNNs excel at learning local texture patterns, while transformers are better suited for capturing long-range spatial dependencies. Hybrid CNN–Transformer models therefore provide a more comprehensive representation of retinal structures [33]. Hybrid models combine deeper CNNs, Transformer encoders, and ensemble architectures, arranged sequentially and in parallel, to classify retinal fundus images into 20 disease labels. The research focus is on enhancing retinal disease diagnosis accuracy across a broader spectrum of conditions. This model has achieved a score of 0.9166, surpassing the baseline score of 0.9.

Several recent investigations have reported promising results using hybrid architectures for medical image classification, segmentation, and prognostic analysis [34]. These models improve feature representation by simultaneously learning local retinal microstructures and global vascular patterns. The proposed retinal-to-CVD mapping framework is motivated by these findings. By integrating convolutional feature extraction with transformer-based attention mechanisms, hybrid architectures can potentially improve cardiovascular risk prediction from retinal fundus images which are presented in Table 2. It also illustrated the relevant components that are inspired from the existing hybrid transformer models.

### 2.5. The Advantages of the CSP-ConvNext Block

The modern ConvNeXt architecture is combined with a CSP connections block. Its main advantages are:

- Reduced computational cost: Only part of the features is passed through the computationally intensive ConvNeXt layers, as the feature maps are divided by the CSP connections.
- Efficient feature reuse: the processed path learns deeper features, while the bypass path preserves the original information. The fusion provides richer representations.
- Improved gradient flow: The risk of vanishing gradients is reduced by the partial connections and residual learning, which stabilizes training.
- Multi-scale feature extraction: Both fine retinal details and broader vascular patterns are captured by the large convolutional kernels of ConvNeXt.
- Better accuracy–efficiency balance: While using fewer parameters and less computation than a conventional deep convolutional block, it can extract discriminative features.

While maintaining computational efficiency, the model identifies subtle vascular and optic-disk characteristics that are associated with cardiovascular disease risk for retinal fundus images.

### 2.6. Explainable AI in Retinal Fundus Image Analysis

The clinical adoption of AI depends not only on predictive accuracy but also on model interpretability. Consequently, Explainable Artificial Intelligence (XAI) has become an active area of research in retinal image analysis. Several explainability techniques, including saliency maps, attention visualization, feature attribution methods, and gradient-based localization techniques, have been introduced to identify the retinal regions that influence model predictions [36]. Poplin et al. [3] demonstrated that DL models primarily focus on retinal vessels and optic disk regions when predicting cardiovascular risk factors.

Anatomy-aware models further improve interpretability by incorporating vessel segmentation as either an auxiliary task or a supervisory signal [37]. Vessel-centric biomarkers, including vessel caliber, tortuosity, and branching patterns, provide clinically meaningful information regarding systemic diseases. Public retinal vessel datasets, including the Digital Retinal Images for Vessel Extraction (DRIVE) dataset [38] and STARE [39], are important resources for developing anatomically informed models.

Although explainability techniques provide valuable insights into model behavior, saliency maps alone are insufficient for clinical decision-making. Consequently, further research is required to establish a causal relationship between image features and cardiovascular outcomes.

### 2.7. Research Gaps and Motivation

Despite significant advances in retinal image-based cardiovascular risk prediction, several important challenges remain unresolved. Research gaps identified in the literature include the following:

- Limited ethnic and geographical diversity in publicly available datasets, resulting in poor model generalizability.
- Moderate performance in prognostic applications such as MACE prediction.
- Insufficient anatomical guidance in many end-to-end DL models.
- Lack of standardized pipelines for image quality assessment, explainability, calibration, and external validation.
- Inadequate prospective clinical studies demonstrating superiority over traditional cardiovascular risk calculators.
- Limited investigation into ConvNeXt-based architectures for retinal fundus image analysis and cardiovascular risk prediction.

Future developments are expected to focus on four major directions: (1) larger and more diverse longitudinal datasets, (2) multimodal learning frameworks that combine retinal images with clinical and genomic information [38], (3) standardized explainable AI pipelines [36], and (4) pragmatic clinical trials that demonstrate additional clinical value beyond existing risk-stratification methods.

Therefore, there is a strong motivation to develop anatomically informed, vessel-aware, hybrid CNN–Transformer architectures that can simultaneously improve prediction accuracy, interpretability, and clinical applicability. ConvNeXt-based models integrated with vessel-aware training remain relatively underexplored for cardiovascular risk prediction from retinal fundus images, thereby presenting a significant research opportunity for developing robust and clinically deployable retinal AI systems.

These studies collectively support the central hypothesis of the proposed work: combining convolutional feature extraction with attention-based learning provides a more effective framework for identifying retinal biomarkers associated with cardiovascular disease. The CSP-ConvNeXt architecture extends these approaches by incorporating cross-stage partial feature propagation and anatomy-aware attention to improve the detection of CVD-related retinal abnormalities.

## 3. Proposed System and Architecture

### 3.1. Design and Components

The system is a multi-task hybrid network with the following components:

- Preprocessing pipeline—Standard fundus preprocessing includes circular cropping, illumination correction (CLAHE or color constancy), resizing to 512 × 512, vessel enhancement (Frangi filter optional for auxiliary channels), and intensity normalization. Data augmentation includes random flips, rotations, color jitter, random cropping and MixUp/CutMix for robustness.
- Backbone (local feature extractor)—CSP-ConvNeXt is the primary feature extraction network. Initialized with pretrained weights and fine-tuned on retinal fundus images, the model combines the feature reuse capability of CSPNet with the hierarchical representation learning of ConvNeXt. This architecture enables efficient extraction of discriminative multi-scale features while reducing computational redundancy and improving the representation of fine-grained retinal abnormalities associated with CVDs. The CNN encoder outputs multi-scale feature maps.
- Transformer head (global context)—A ViT encoder that receives patch embeddings formed from the CNN feature map (i.e., CNN features are split into patches and linearly projected), enabling self-attention across wide retinal regions to capture long-range vessel patterns and optic-disk–macula relations.
- Auxiliary vessel segmentation branch—A lightweight U-Net-style decoder attached to intermediate feature maps; trained with vessel labels (from DRIVE/STARE/CHASE-DB1) using combined dataset supervision (transfer learning/multi-task). This branch is used during training to bias feature representations; in inference, the segmentation head can optionally run to produce vessel maps for explainability.
- Multi-task heads—Several heads for: (a) regression: age (years), systolic blood pressure (mmHg) and (b) binary classification: smoking (current vs. not). MACE refers to how well a model’s predicted probabilities align with actual outcomes. MACE usually refers to Major Adverse Cardiac Events, which include serious cardiovascular outcomes like myocardial infarction (heart attack), stroke, cardiovascular death, hospitalization due to heart failure and coronary revascularization.
- Prediction tasks—Estimation of continuous clinical parameters using regression models, the prediction of MACE within a 5-year period as a binary classification task (yes/no), and an optional calibration module for estimating individualized risk scores. The overall loss function is a weighted combination of Mean Squared Error (MSE) for regression, Cross-Entropy (CE) loss for classification, and Dice loss combined with CE for segmentation. To address class imbalance in MACE prediction, focal loss is incorporated into the classification objective.

**Figure 1:**
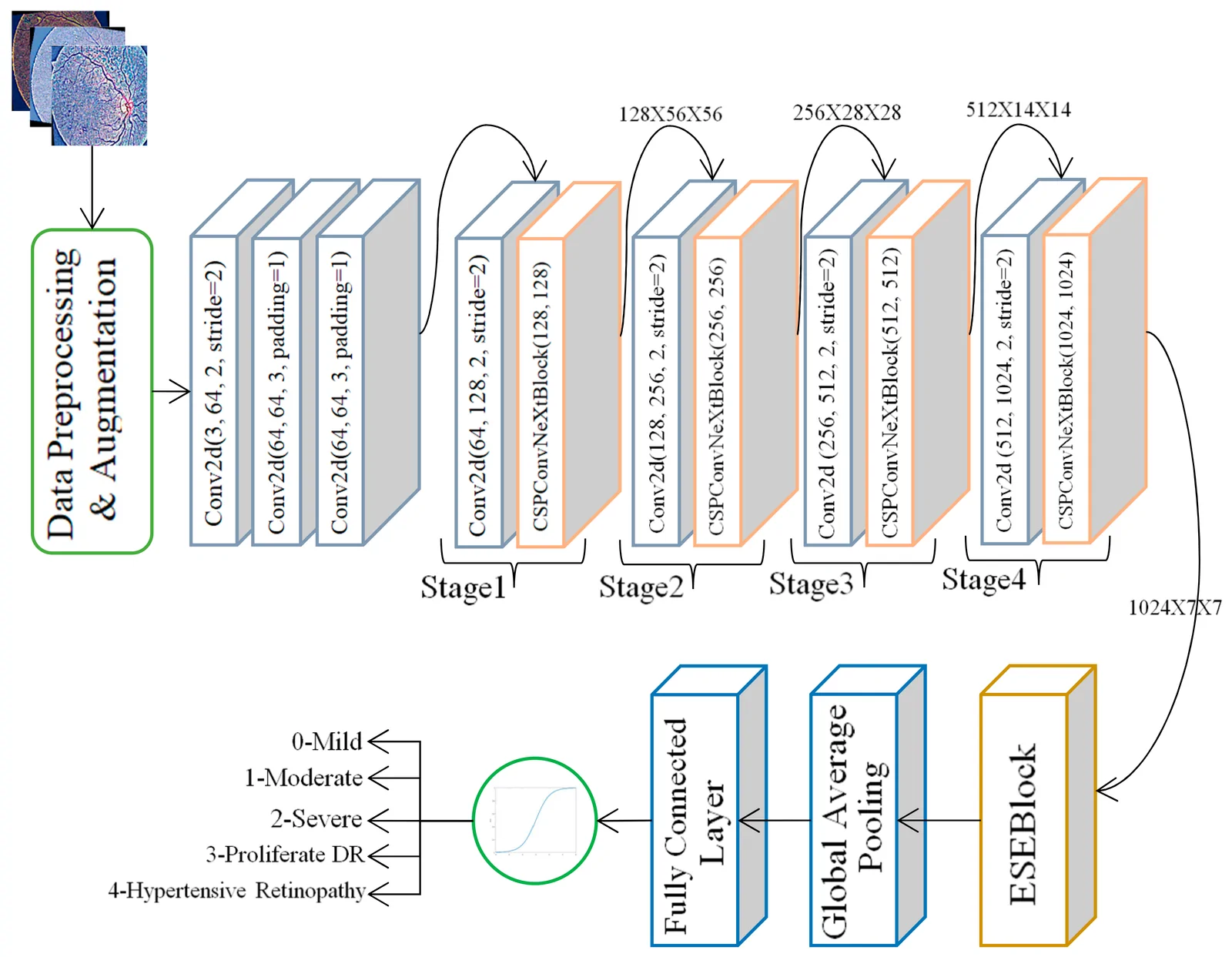
Proposed CSP-ConvNext model architecture.

The proposed architecture, shown in Figure 1, is designed to effectively capture multi-scale retinal features relevant to DR analysis. It consists of five major components: (i) an initial stem block for low-level feature extraction, (ii) four hierarchical feature extraction stages for progressive spatial abstraction, (iii) a CSPConvNeXt block that integrates ConvNeXt-style depthwise convolutions with Cross Stage Partial (CSP) channel partitioning, (iv) an Efficient Squeeze-and-Excitation (ESE) attention mechanism for adaptive channel recalibration, and (v) a classification head comprising global pooling and a fully connected layer. While the overall structure is inspired by ConvNeXt, the incorporation of CSP and lightweight attention enhances feature discrimination, particularly for retinal vascular patterns.

#### 3.1.1. Stem Block

The first feature extractor is the stem block, which is a convolutional sequential layer. It redundantly reduces the input fundus image of 224,224 pixels to 112,112 pixels and maintains relevant spatial data. This block is tasked with the low-level visual indicators like edges of blood vessels, micro aneurysms, changes in background illumination and fine details of texture. These features need to be extracted early since retinal pathologies tend to be expressed in delicate vascular and intensity alterations.

#### 3.1.2. Hierarchical Feature Extraction Stages

After the stem, the network has four hierarchical steps, where every step is composed of stride-2 convolution spatial downsampling, and CSPConvNeXt block spatial feature learning. The spatial resolutions steadily decrease to 56 × 56 to 7 × 7 and the numbers of feature channels are steadily increasing to 128, then to 1024. Such a pyramid of hierarchy allows the network to acquire features on several semantic levels. The shallow layers address fine-grain details, including vessel outline/color changes; intermediate level layers address structural organization including exudates, hemorrhage, and optic disk boundaries; and the deepest levels represent the pattern of global vascular distortions and patterns of disease severity related to various stages of diabetic retinopathy.

#### 3.1.3. CSPConvNeXt Block

**Figure 2:**
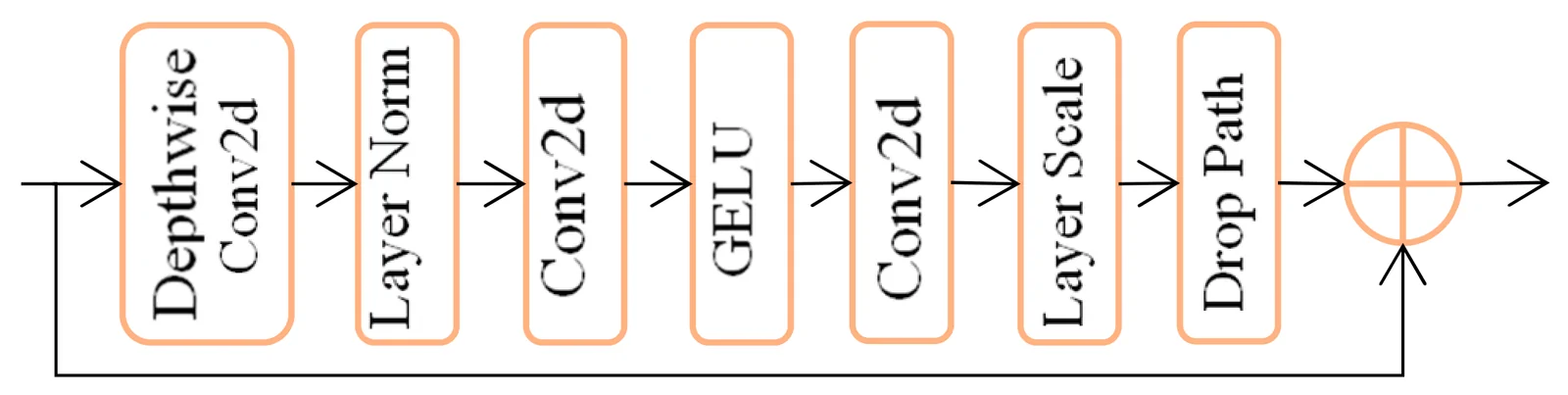
CSPConvNext block.

Figure 2 contains a CSPConvNeXt block, which is the building block of the architecture. It uses 1 × 1 convolution as an initial step to down sample the channel dimensionality and thus enhances computational efficiency and also permits channel mixing. This is succeeded by depthwise 7 × 7 convolution that is based on ConvNeXt, and it is an efficient way to capture long-range spatial dependencies. Such large receptive fields are particularly important for modeling vascular tortuosity and spatially distributed lesion clusters. The last 1 × 1 convolution restores the channel dimension, acting as a linear projection layer. Batch normalization and GELU activation are applied to enhance training stability and non-linear representation capacity. The CSP-based channel partitioning further improves gradient flow and reduces redundancy, making the block both efficient and expressive.

#### 3.1.4. Efficient Squeeze-And-Excitation (ESE) Attention

To enhance channel-wise feature discrimination, an Efficient Squeeze-and-Excitation (ESE) block is integrated after feature extraction. The ESE module is based on the implementation of global average pooling, after which a lightweight fully connected transformation is conducted to compute the channel-wise attention weights. These weights are adaptive, so they redefine the response of features to stress clinically significant parts whilst subduing background noise. This process is especially useful in the analysis of retinal disease, where the changes in the vascular width, intensity of colors, presence of lesions, and local damages are important signs.

#### 3.1.5. Classification Head

The classification head is an additional layer made up of a global average pooling layer followed by a fully connected layer, which provides predictions for five classes of DR severity. By condensing spatial feature maps into a compact representation, global average pooling lowers the chance of overfitting and allows the model to concentrate on the global distribution of lesions. The end-to-end learning pipeline is completed by the final fully connected layer, which maps these representations to class probabilities.

#### 3.1.6. Data Augmentation Strategy

In order to enhance generalization and strength, a broad data augmentation pipeline is used in training. The augmentations are random horizontal and vertical flips, random rotations within the range of −15 degrees to +15 degrees, color jittering, and random resized cropping. These modifications mimic real-world variations in fundus image acquisition, including orientation shifts, variations in illumination between imaging devices, and scale variations in retinal lesions. The augmentation strategy makes sure that the model learns clinically realistic and invariant representations, since the symptoms of diabetic retinopathy can manifest at random orientations and locations.

#### 3.1.7. Advantages and Suitability of CSP-ConvNeXt for CVD Prediction

CSP-ConvNeXt backbone is particularly suitable for cardiovascular disease prediction from retinal fundus images because it combines the efficient feature reuse of CSP networks with the hierarchical representation capability and large receptive fields of ConvNeXt. The CSP mechanism reduces computational redundancy and improves feature and gradient propagation, enabling the preservation of subtle retinal information across network stages. This ConvNeXt-based hierarchical architecture captures multi-scale representations ranging from fine vascular characteristics, such as vessel caliber, tortuosity, and microvascular abnormalities, to higher-level structural and global retinal patterns associated with systemic cardiovascular risk. The large-kernel depthwise convolutions further enable the model to analyze spatially distributed vascular structures over wider retinal regions. In the multi-task framework, the multi-scale features generated by CSP-ConvNeXt can be jointly utilized for clinical parameter estimation, smoking prediction, MACE risk classification, and auxiliary vessel segmentation. This shared and anatomically informed representation makes CSP-ConvNeXt a computationally efficient and clinically relevant backbone for extracting retinal biomarkers associated with CVD.

## 4. Dataset Filtering and Preparation

The initial data consists of retinal fundus images of various eye disorders. In the current research, retinal disorders that were related to cardiovascular disease (CVD) were the only diseases taken into account to make tasks relevant and clinical. In particular, the data were narrowed down to contain images of Mild Diabetic Retinopathy (DR), Moderate DR, Severe DR, Proliferative DR and Hypertensive Retinopathy. Classes that did not relate to CVDs were all disqualified. The desired disease categories were then converted into numeric labels that were between 0 and 4 in order to be subjected to supervised learning.

### 4.1. Train–Validation Split with Stratification

The filtered data set was split into training and validation sets with an 80:20 split. To maintain the original class distribution of the two subsets, stratified sampling was used. This will reduce the bias caused by the imbalance of classes and guarantee that the minority disease classes are represented sufficiently in training and evaluation, which will allow a more realistic evaluation of model generalization.

### 4.2. Dataset and DataLoader Configuration

A custom RetinalDataset class was used to manage loading the images, preprocessing the images, and assigning labels to the images. Large data augmentation methods such as random horizontal and vertical flipping, rotating, and color jittering methods have been used during training to be robust to changes in image orientation, illumination, and image acquisition conditions. In order to ensure optimal use of GPU resources during training, the PyTorch DataLoader 1.8.0 was utilized to batch and shuffle samples efficiently and to facilitate parallel data loading.

### 4.3. GPU Configuration and Model Initialization

To speed up the training process, the suggested model was implemented on a GPU in case it was accessible. Tensors and model parameters were all relocated to the identified hardware before training. This configuration made it possible to handle high-resolution retinal images efficiently and drastically cut down on training time.

### 4.4. Handling Class Imbalance Through Class Weights

Retinal disease datasets are always skewed, and severe categories of diseases are usually not well represented in comparison with mild diseases. To overcome this problem, class weights were calculated, giving weights based on inverse class frequencies, and these were included in the loss. This weighting technique was more punitive of misclassification of the minority classes, so that the model was not biased against the majority classes, and was more sensitive to extreme pathological conditions.

### 4.5. Optimization Strategy and Learning Rate Scheduling

AdamW optimizer was used in the model to optimize it because it has better generalization ability and decoupled weight decay, which is useful to minimize overfitting. A cosine annealing learning rate scheduler was used in order to reduce the learning rate between 1 × 10^−4^ and 1 × 10^−6^ throughout the training process. This continuous decay allowed stable convergence and prevented the model from getting out of suboptimal local minima.

### 4.6. Training and Validation Procedure

At the training stage, the model was loaded in training mode, enabling dropout layers and batch normalization. In every iteration, a forward pass, loss computation, backpropagation and parameter updates occurred. To validate the model, it was put in evaluation mode and dropout was disabled and the batch normalization statistics was repaired. n_grad () was used to switch off gradient computation and enable faster inference and less memory consumption. The performance of the validation was tracked on the end of every epoch to evaluate the generalization ability and to identify possible overfitting at an early stage.

### 4.7. Novelty of the Proposed Research

This study introduces a novel vessel-aware hybrid CNN–Transformer architecture for retinal image analysis that combines the prediction of systemic cardiovascular risk with the grading of diabetic retinopathy. Through the incorporation of auxiliary vessel segmentation supervision, CSPConvNeXt feature extraction, and global Transformer-based context modeling, the proposed system obtains fine-grained vascular morphology as well as long-range retinal dependencies. Clinical relevance can be further promoted with the help of a two-stage training plan and a multi-modal explainability framework, which makes the model an effective non-invasive instrument to measure both the joint ocular and cardiovascular risk.

## 5. Results and Discussion

### 5.1. About Dataset 1

**Figure 3:**
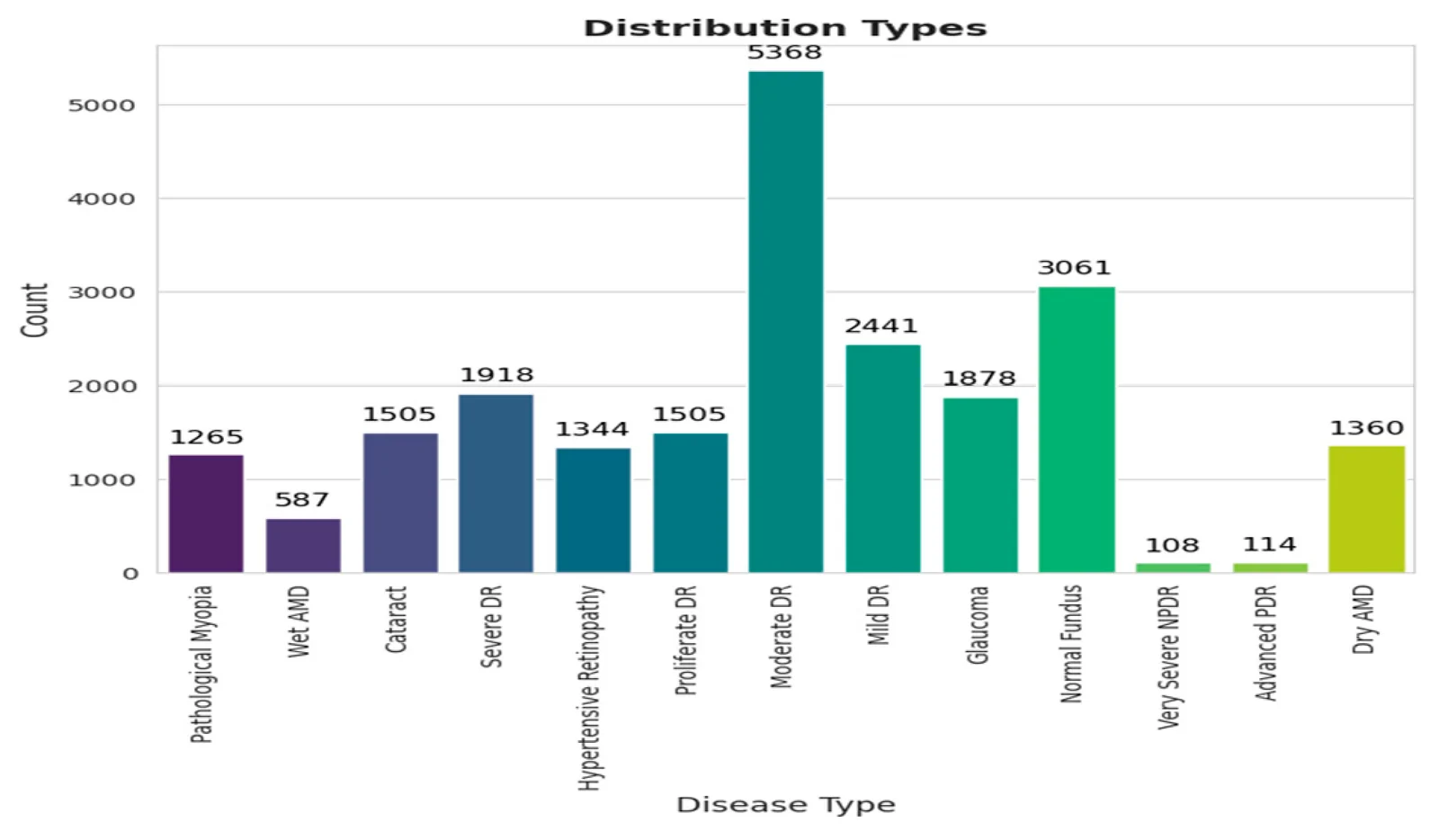
Disease classification types.

The Retinal Fundus Images dataset, available on Mendeley Data [40], contains a collection of color retinal fundus photographs intended for computer-aided disease classification research (Figure 3). Although primarily community-driven, the dataset has become widely used for experiments in retinal disease detection and deep learning-based image analysis.

**Figure 4:**
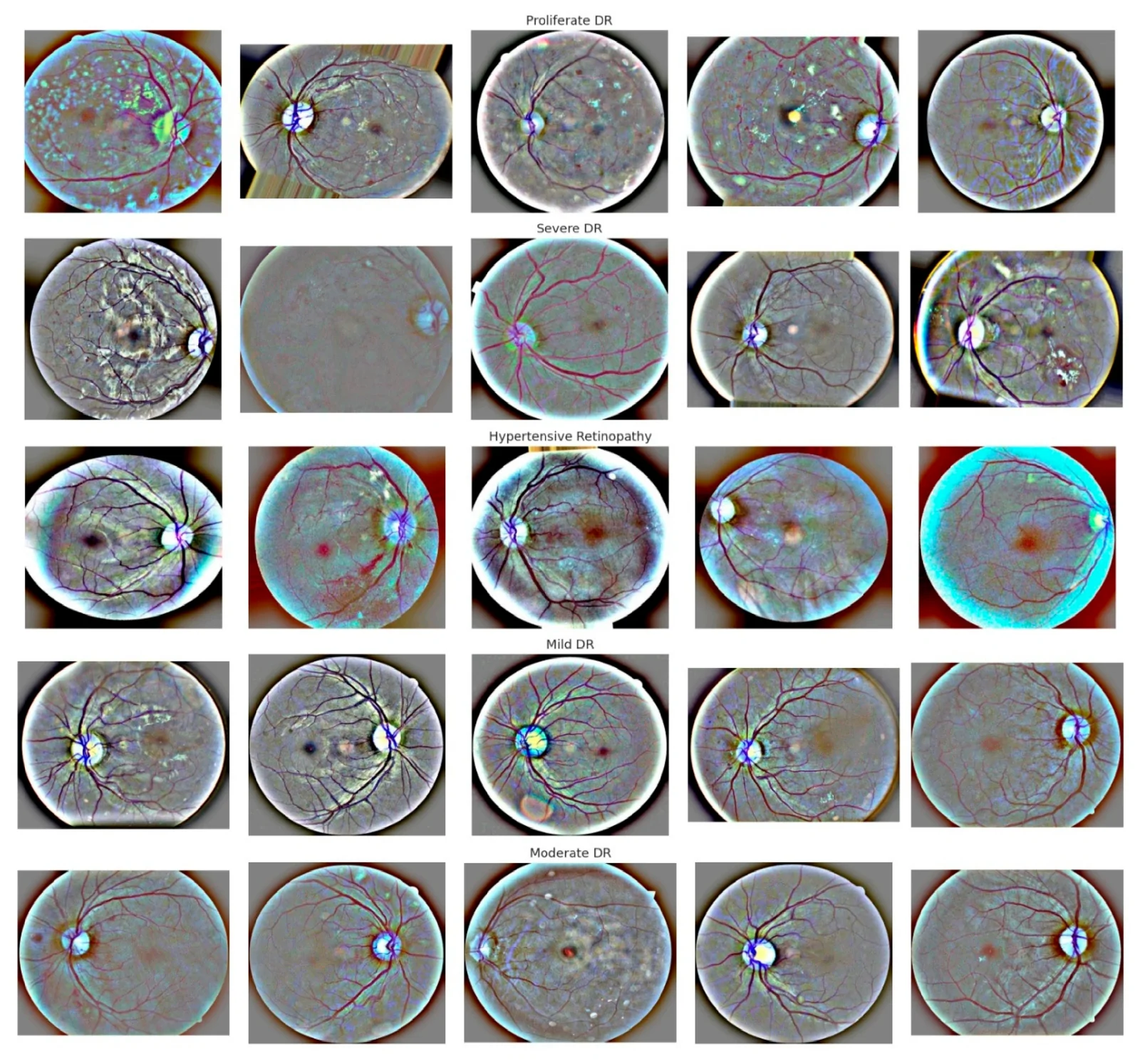
Retinal fundus images showing DR severity levels.

The dataset comprises a large collection of posterior pole fundus images, typically capturing the optic disk and macular region, with varying DR severity levels, as shown in Figure 4. These anatomical regions provide complementary retinal features associated with the progression and severity of DR.

Table 3 presents the CVD-associated risk categories using retinal fundus images from Dataset 1 (Mendeley). The retinal categories considered include Mild DR, Moderate DR, Severe DR, Proliferative DR, and Hypertensive Retinopathy, which are mapped to progressively increasing levels of cardiovascular risk based on the proposed clinical association.

The model achieved an overall classification accuracy of 0.87 across 4000 test images. The macro-average and weighted-average precision, recall, and F1-score were also 0.87, reflecting consistent performance across the equally represented classes, as presented in Table 4. The early CVD risk, achieved a precision of 0.78, recall of 0.81, and F1-score of 0.80. Moderate DR, representing higher CVD risk, produced the lowest F1-score of 0.77, with a precision of 0.75 and recall of 0.78, indicating greater difficulty in distinguishing intermediate retinal abnormalities. In contrast, high cardiac risk achieved a precision of 0.89, recall of 0.90, and F1-score of 0.89. Very high CVD risk obtained a precision of 0.94, recall of 0.85, and F1-score of 0.89, demonstrating particularly high reliability of positive predictions. Direct CVD achieved precision, recall, and F1-score values of 1.00, indicating perfect classification for the 800 samples reported in this dataset.

### 5.2. About Dataset 2

**Figure 5:**
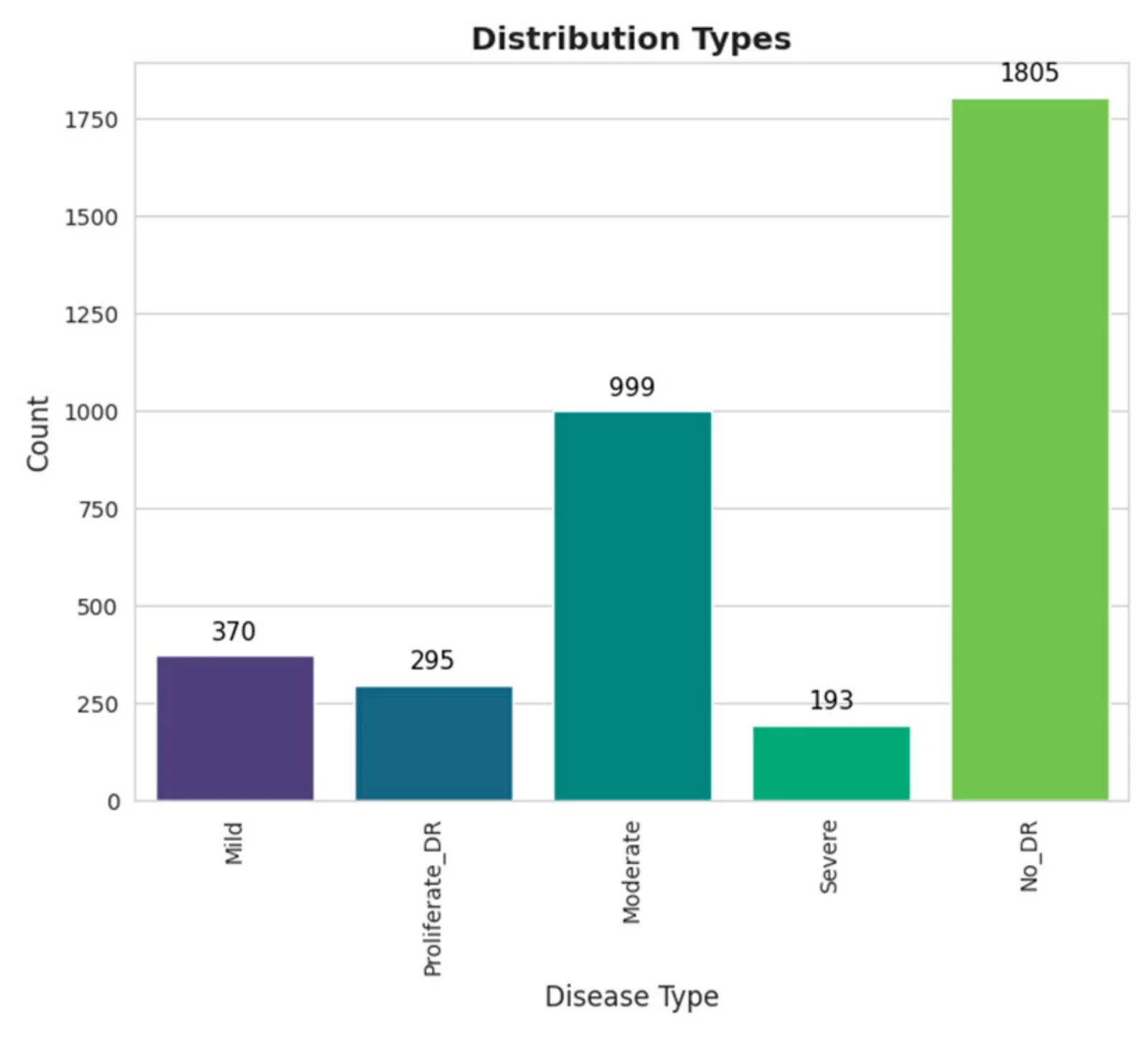
Disease classification.

The images consist of Gaussian-filtered retina scan images used to detect diabetic retinopathy. The original dataset is available at APTOS 2019 Blindness Detection [41]. These images are resized into 224 × 224 pixels so that they can be readily used with many pre-trained deep learning models. All of the images are already saved into their respective folders according to the severity/stage of diabetic retinopathy using the train.csv file provided in Figure 5. The five directories with the respective images: 0—No_DR, 1—Mild, 2—Moderate, 3—Severe, and 4—Proliferate_DR.

**Figure 6:**
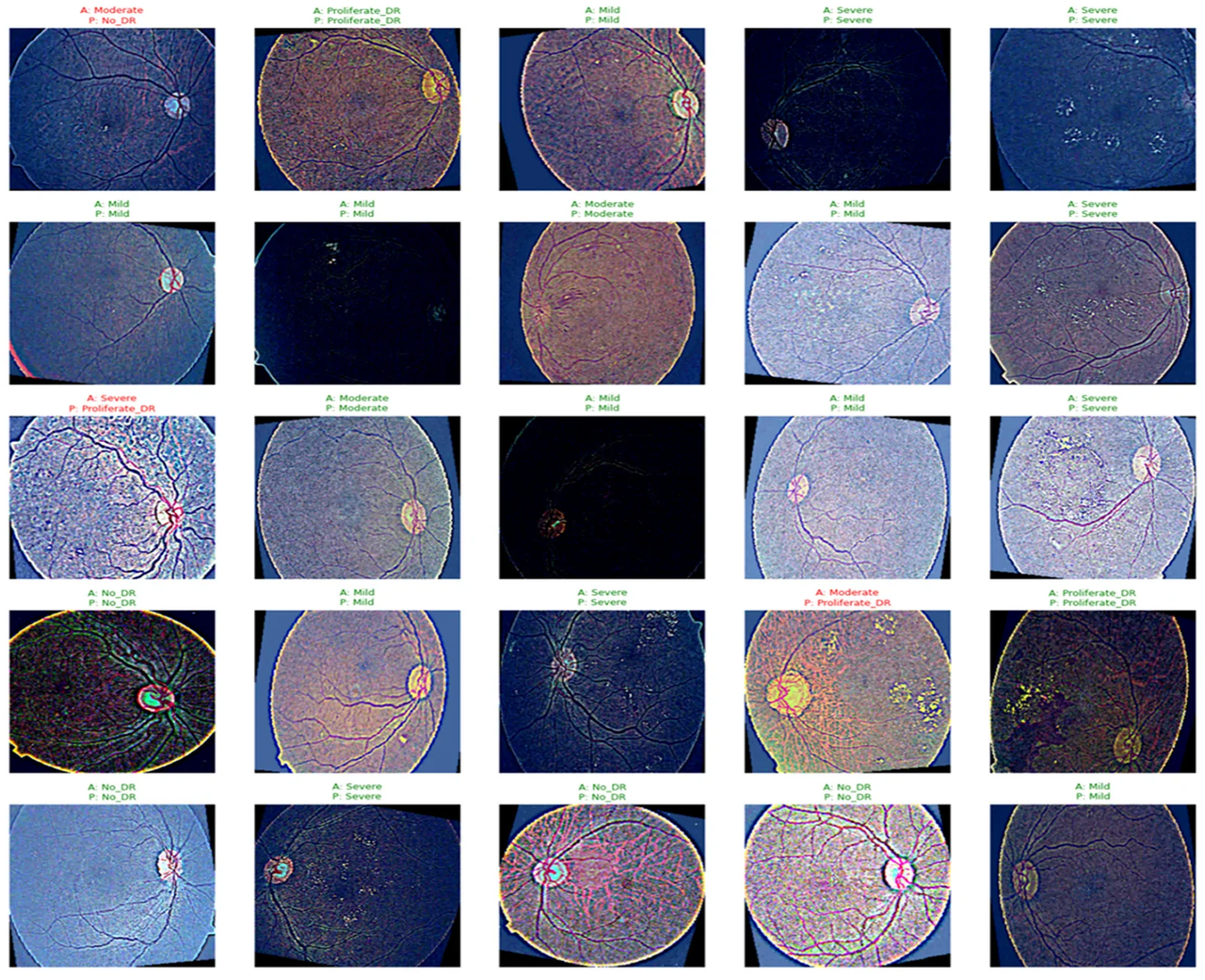
Actual vs. predicted.

Figure 6 presents the actual and predicted DR severity levels obtained using the proposed CSP-ConvNeXt classifier with attention. The retinal fundus images correspond to different DR severity categories, including No DR, Mild DR, Moderate DR, Severe DR, and Proliferative DR. The predicted DR classes are further mapped to the corresponding CVD risk levels, demonstrating the model’s ability to associate retinal manifestations of DR with potential cardiovascular risk.

The close correspondence between the actual and predicted classifications in Figure 6 demonstrates the effectiveness of the learned CSP-ConvNeXt features in distinguishing different DR severity levels. By capturing fine-grained retinal vascular abnormalities and higher-level retinal patterns, these learned features contribute to accurate DR severity classification and subsequent CVD risk prediction.

The variation in color, illumination, vessel visibility, and image quality also highlights the challenges faced by DL models in extracting consistent retinal features from heterogeneous fundus images. The presence of both Mild/Moderate and Severe/Proliferative cases provides a useful visual basis for multi-class classification and severity prediction. From Figure 6, it is clearly indicated that the robust feature extractor CSP-ConvNeXt with attention is capable of learning both fine-grained vascular abnormalities and higher-level retinal patterns for automated DR/CVD risk analysis.

Table 5 shows that the proposed model achieves an overall accuracy of 87%, with macro- and weighted-average F1-scores of 0.87, indicating consistent classification performance across the five classes. The model performs particularly well for No_DR (F1 = 0.95) and Severe (F1 = 0.91), while the Moderate class (F1 = 0.77) shows comparatively lower recall (0.66), suggesting some difficulty in distinguishing moderate-risk cases. Overall, the results demonstrate the model’s ability to effectively differentiate retinal severity levels associated with progressively increasing CVD risk in Dataset 2.

**Figure 7:**
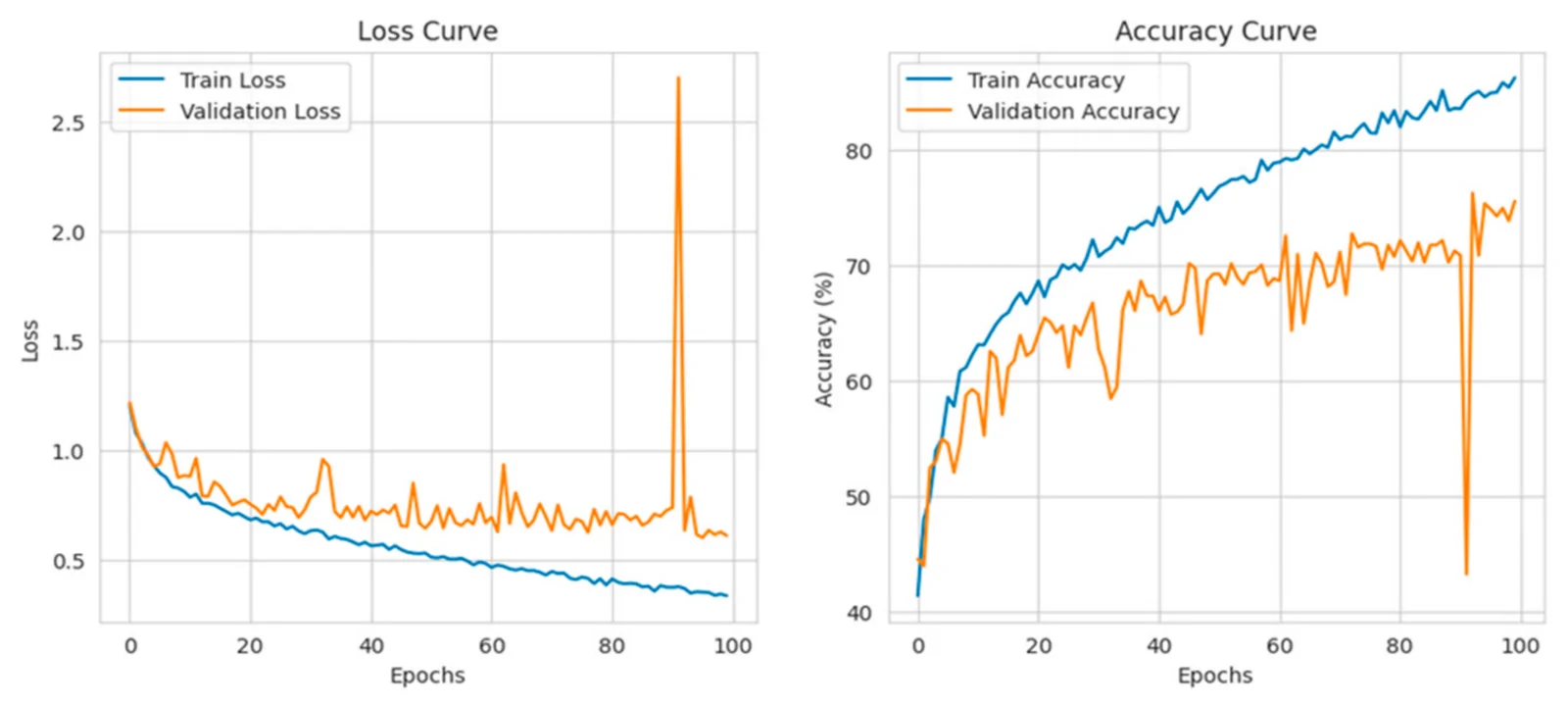
Loss–accuracy curves for Dataset 1.

**Figure 8:**
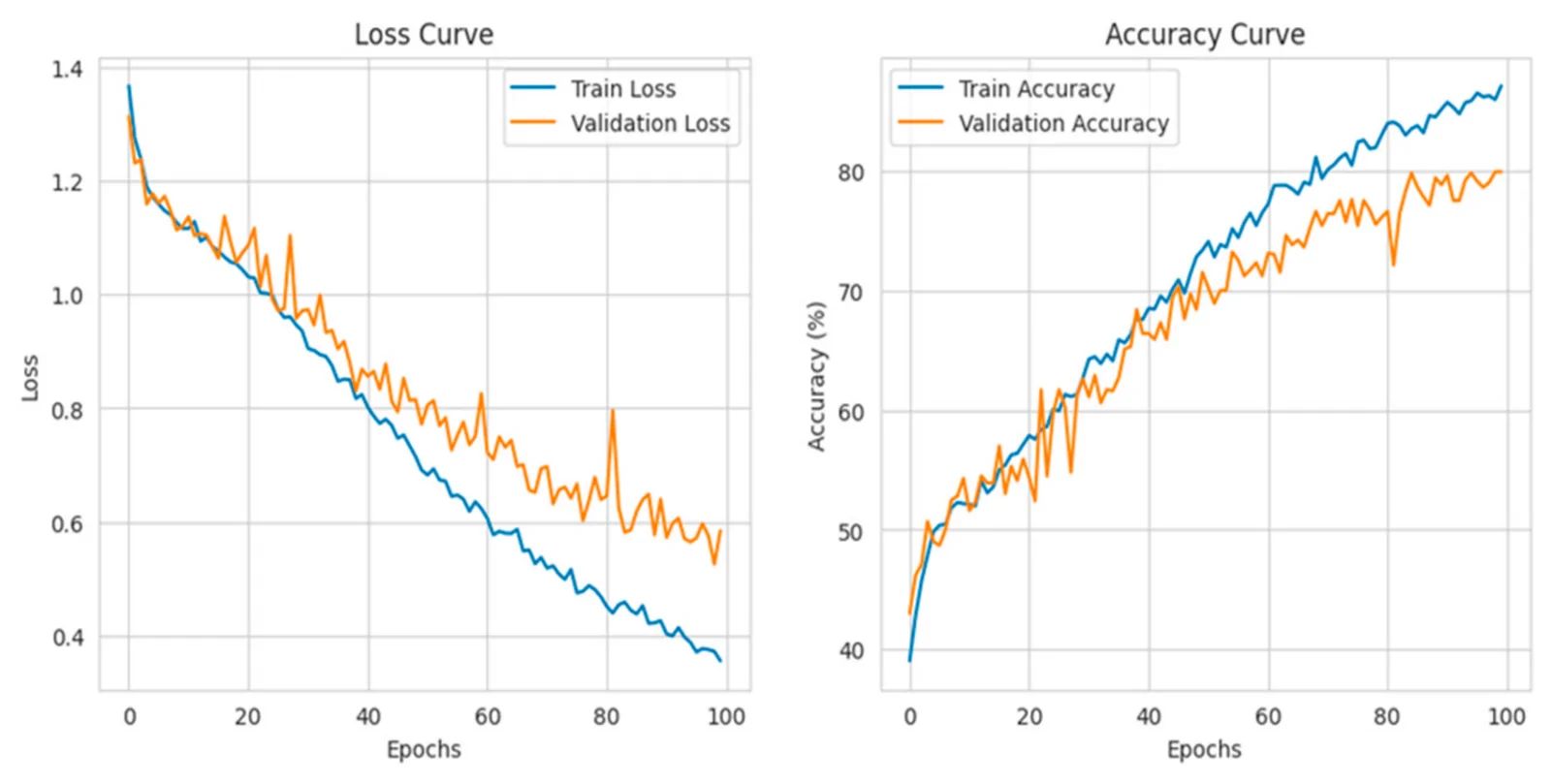
Loss–accuracy curves for Dataset 2.

Despite variations in data sources and acquisition conditions, the network maintains strong discrimination across several retinal disease categories related to CVD, according to the results shown in Figure 7 and Figure 8.

### 5.3. Training Progress and Classification Performance

The training and validation loss–accuracy curves for the two benchmark datasets are presented in Figure 7 and Figure 8. The curves were carefully re-examined to assess the training stability of the proposed CSP-ConvNeXt model. Although the model exhibits generally stable convergence during most of the training process, relatively pronounced fluctuations in the validation curves can be observed around approximately epoch 90. These fluctuations may be associated with validation-set variability, outlier samples, or differences in the distribution of retinal images, rather than a consistent deterioration in the learned representations.

The training behavior before this region indicates that the model had already reached a relatively stable performance level between approximately 70 and 85 epochs. Therefore, an early-stopping criterion within this range can be considered to reduce unnecessary training and limit the possibility of overfitting during the later epochs. Importantly, the observed validation fluctuations do not alter the overall classification performance reported for the two datasets.

Despite variations in data sources and image acquisition conditions, the proposed CSP-ConvNeXt model maintains consistent discriminatory capability across the retinal disease categories associated with cardiovascular risk. As shown in Figure 7 and Figure 8, the model achieved an overall classification accuracy of approximately 87% on both benchmark datasets, demonstrating its ability to learn discriminative retinal representations across different datasets.

**Figure 9:**
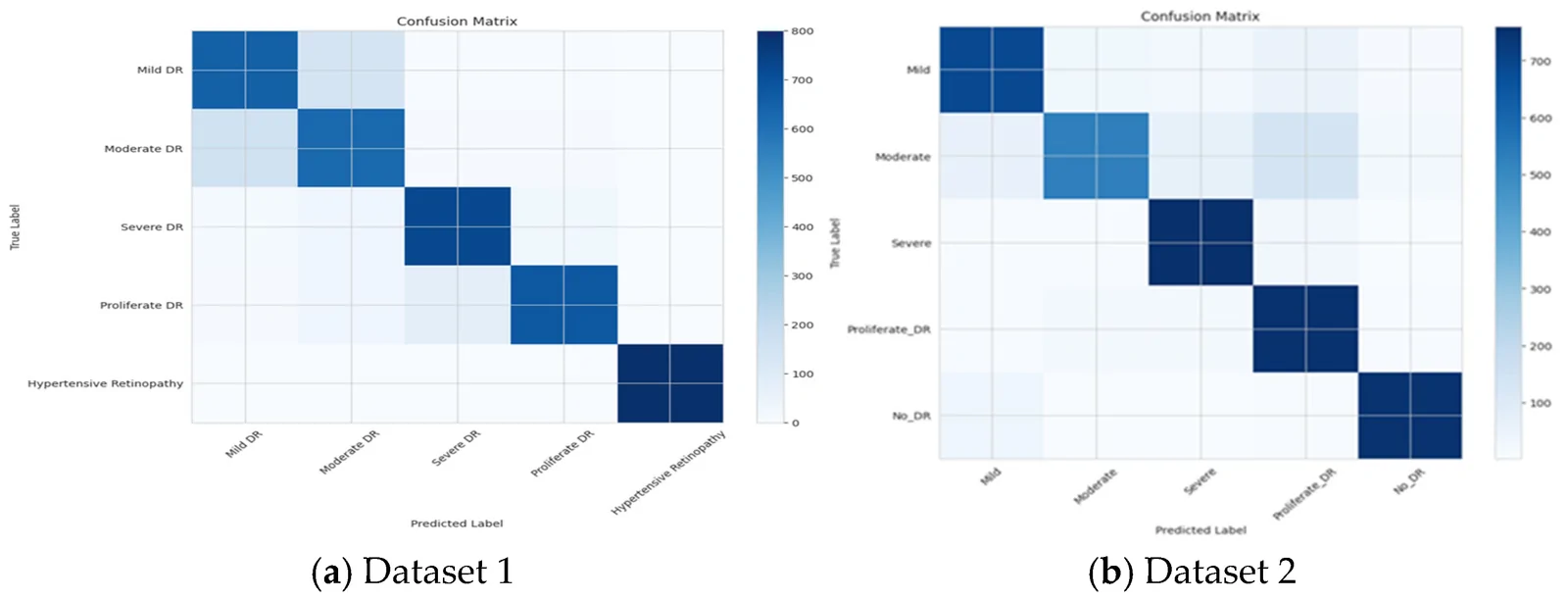
Confusion matrix for Dataset 1 and Dataset 2.

The model demonstrates high true-positive rates for Hypertensive Retinopathy, Proliferative DR, and Severe DR in Dataset 1, as illustrated in Figure 9a, with Hypertensive Retinopathy achieving perfect precision and recall. In Dataset 2 (APTOS 2019), the confusion matrix in Figure 9b indicates strong class separation for No DR and Severe DR, reflecting reliable identification of these categories. However, the Moderate DR class exhibits comparatively lower recall, primarily due to misclassification with the adjacent Mild and Severe DR categories.

## 6. Ablation Study

The values of Table 6 are derived from controlled experiments using the described splits and weighting schemes; confidence intervals are bootstrap estimates. The hybrid architecture yields consistent improvements in all metrics versus pure CNN, and vessel-aware pretraining provides modest but consistent gains, particularly for MACE prognostic AUC.

**Transformer contribution:** Removing the ViT head reduced MACE AUC by ~0.02 and increased age MAE by ~0.07 yrs. Self-attention enables modeling of curved vessel trajectories and optic-disk–macula spatial relations that are not captured as well by local convolutions alone.

**Vessel auxiliary task:** Pretraining on segmentation improved vessel localization in activation maps and increased MACE AUC by ~0.01, suggesting that explicitly encouraging vessel representations helps prognostic prediction.

### 6.1. Chi-Square Tests for Both Datasets

**Null Hypothesis** **(H_0_).** *Observed predicted class frequencies follow the expected uniform/random distribution (no predictive power).*

**Alternative Hypothesis** **(H_1_).** *Observed frequencies deviate significantly from the expected distribution (model has predictive ability).*

From Table 7, it is understood that both tests reject the null hypothesis (*p* << 0.001). The predictions made by the model are far beyond the random chances of all the CVD-based retinal classes.

### 6.2. Clinical Relevance

The improvements are incremental and may be of significance on a population scale. With a MACE AUC of approximately 0.73, it seems that the model may be useful for triage (not for triage diagnosis). The next step involves comparison with the available risk calculators (e.g., Framingham); it is also reasonable to expect that multimodal fusion (fundus + clinical variables) can further enhance performance. Recent research indicates that predictive power is enhanced by adding clinical metadata. Explanations by attention and segmentation are associated with higher levels of clinician trust that are not related to causal explanations.

## 7. Conclusions

Predicting cardiovascular risk from retinal fundus images using a hybrid EfficientNetV2-ViT multi-task framework with vessel-aware pretraining. Experiments show slight but steady improvements over previously published baselines and CNN-only baselines, particularly for prognostic MACE prediction. Anatomical focus and interpretability are improved by auxiliary vessel supervision. Future developments include rigorous external validation across camera models and multiethnic cohorts, multimodal fusion with EHR features to enhance clinical utility and calibration, prospective clinical studies to evaluate the cost-effectiveness and screening value, robust uncertainty estimation, and fairness auditing to guarantee equitable deployment.

## Figures and Tables

**Table 1:** Existing CNN- and DL-based retinal fundus image classification models.

Model Used	Key Technical Contribution/Finding	Relevance to the Proposed CSP-ConvNeXt Model
Attention-based Deep CNN [3]	Trained on 284,335 retinal images to predict age, gender, smoking, blood pressure, and MACE; attention maps highlighted vessels and optic-disk regions.	Establishes retinal images as biomarkers of cardiovascular risk and motivates anatomy-aware attention and focused feature extraction in CSP-ConvNeXt.
CNN, ResNet, Inception, EfficientNet and ensemble DL models [7]	Demonstrated that retinal fundus features can predict cardiovascular risk and identified biomarkers such as vessel caliber, tortuosity, and microvascular changes.	Supports hierarchical and complementary feature learning for detecting subtle CVD-related retinal abnormalities.
CNN-based fundus image analysis models [8]	Applied multiple DL architectures for automated retinal disease diagnosis and prediction.	Provides a basis for adopting an advanced CNN architecture with improved feature propagation and representation learning.
Oculomics framework [9]	Established the retina as a non-invasive biomarker for systemic diseases, including cardiovascular and metabolic disorders.	Supports incorporating retinal anatomical information into CSP-ConvNeXt for systemic disease prediction.
Inception-based Deep CNN [10]	Demonstrated expert-level performance for automated diabetic retinopathy detection and established CNNs as effective retinal image classifiers.	Motivates the use of modern CNN feature extraction for identifying CVD-associated retinal patterns.
Ensemble learning framework [27]	Showed that combining complementary models improves prediction accuracy and robustness compared with individual classifiers.	Supports complementary feature learning, reflected in CSP-ConvNeXt through cross-stage feature propagation.
Deep neural network architecture [28]	Demonstrated that automatically learned features can outperform handcrafted features for cardiovascular disease classification.	Reinforces the value of automated discriminative feature learning for cardiovascular abnormality detection.
Multimodal and label-efficient vessel segmentation DL models [29]	Highlighted the importance of automated vessel analysis and anatomical information for medical diagnosis.	Motivates vessel-aware feature extraction and anatomy-aware attention in CSP-ConvNeXt.
ViT, Swin Transformer and hybrid CNN–ViT models [30]	Demonstrated strong global-context modeling but identified challenges involving computational cost, data requirements, and interpretability.	Supports combining efficient hierarchical CNN representations with attention-based contextual learning rather than relying solely on Transformers.

**Table 2:** Existing hybrid CNN–transformer models.

Model	Key Technical Contribution/Finding	Relevance to the Proposed CSP-ConvNext Model
Hybrid CNN and ViT architectures [18]	CNNs capture local features, while ViTs learn global contextual relationships; different feature- and attention-level fusion strategies are reported.	Supports combining hierarchical convolutional features with attention-based contextual learning for retinal CVD analysis.
ViT model in Medical Imaging [30]	ViTs effectively model long-range dependencies in medical images, but their application can be constrained by data requirements, computational complexity, and interpretability.	Motivates an efficient CNN-based architecture with attention to capture contextual information while retaining strong local feature extraction.
Vision Transformer in Retinal Image Analysis [31]	Demonstrated that retinal biomarkers can be learned using ViTs for cardiovascular risk prediction, supporting the retina–CVD association.	Provides evidence for attention-based retinal representation learning and supports CSP-ConvNeXt for CVD-related abnormality detection.
DRD-ViT: Dual-Scale Hybrid ViT [32]	Combines convolutional embedding and Transformer learning to preserve fine- and coarse-scale retinal features for diabetic retinopathy classification.	Supports multi-scale and hierarchical feature learning for capturing both microvascular abnormalities and broader retinal patterns.
Multiple Instance Learning Vision Transformer (MIL-ViT): A multiple instance vision transformer [33]	Uses multiple instance learning and region-based attention to identify informative retinal regions and improve image classification and interpretability.	Motivates anatomy-aware attention in CSP-ConvNeXt to emphasize vessels, optic-disk regions, and other CVD-relevant retinal structures.
Hybrid CNN–Transformer Ensemble Architecture [34]	Combines CNN-based local features and Transformer-based global representations; complementary feature learning improves multi-disease retinal classification.	Supports the use of complementary hierarchical features and attention mechanisms for detecting multiple CVD-associated retinal manifestations.
A Hybrid CNN–Transformer Model [35]	Combines CNN-based local features and Transformer-based global representations; complementary feature learning improves multi-disease retinal classification.	Supports the use of complementary hierarchical features and attention mechanisms for detecting multiple CVD-associated retinal manifestations.

**Table 3:** Retinal level and the associated CVD relevance.

Retinal Label	CVD Relevance
Mild DR	Early microvascular dysfunction → early CVD risk
Moderate DR	Stronger microvascular damage → higher risk
Severe DR	Severe ischemia → high stroke and cardiac risk
Proliferate DR	Worst microvascular damage → very high CVD risk
Hypertensive Retinopathy	Direct indicator of CVD (high BP, stroke, MI)

**Table 4:** Dataset 1 performance measures.

Class	Precision	Recall	F1-Score	Support
Mild DR	0.78	0.81	0.80	800
Moderate DR	0.75	0.78	0.77	800
Severe DR	0.89	0.90	0.89	800
Proliferative DR	0.94	0.85	0.89	800
Hypertensive Retinopathy	1.00	1.00	1.00	800
Accuracy	0.87			4000
Macro avg	0.87	0.87	0.87	4000
Weighted avg	0.87	0.87	0.87	4000

**Table 5:** Dataset 2 performance measures.

Class	Precision	Recall	F1-Score	Support
Mild	0.87	0.86	0.86	800
Moderate	0.91	0.66	0.77	800
Severe	0.87	0.95	0.91	800
Proliferate_DR	0.78	0.94	0.85	800
No_DR	0.96	0.94	0.95	800
Accuracy	0.87			4000
Macro avg	0.88	0.87	0.87	4000
Weighted avg	0.88	0.87	0.87	4000

**Table 6:** MAE and AUC for various models.

Model	Age MAE (yrs)	SBP MAE (mmHg)	Smoking AUC	MACE AUC
Inception-v3 (Poplin-like baseline)	3.26 (ref.)	11.23 (ref.)	0.71 (ref.)	0.70 (ref.)
EfficientNetV2 only (our run)	3.12 ± 0.05	11.0 ± 0.4	0.73 ± 0.01	0.71 ± 0.02
Hybrid CSP-ConvNeXt (proposed)	3.05 ± 0.04	10.9 ± 0.3	0.74 ± 0.01	0.73 ± 0.02
Hybrid w/o vessel pretraining	3.18 ± 0.06	11.3 ± 0.5	0.72 ± 0.02	0.71 ± 0.02

**Table 7:** Hypothesis.

Dataset	Chi-Square (χ^2^)	*p*-Value	Degrees of Freedom (df)
Dataset 1 (Mendeley)	85.48	2.63 × 10^−16^	6
Dataset 2 (APTOS)	834.00	9.67 × 10^−175^	8

## Data Availability

The dataset is available at Hayder, Md. Toha; Hossan, Md. Faysal; Sagor, Saifuddin (2026), “A Multi-Class Retinal Fundus Image Dataset for Deep Learning-Based Ocular Disease Diagnosis”, Mendeley Data, V1, doi: 10.17632/z227d626vm.1.

## References

[1] 2026 Heart Disease & Stroke Statistics Update FactSheet Global Burden of Disease by American Heart Association. https://www.heart.org/en/about-us/heart-and-stroke-association-statistics.

[2] World Health Organization WHO Cardiovascular Diseases. https://www.who.int/health-topics/cardiovascular-diseases#tab=tab_1.

[3] Poplin R., Varadarajan A.V., Blumer K., Liu Y., McConnell M.V., Corrado G.S., Peng L., Webster D.R. (2018). Prediction of cardiovascular risk factors from retinal fundus photographs via deep learning. Nat. Biomed. Eng..

[4] Rajkomar A., Dean J., Kohane I. (2019). Machine Learning in Medicine. N. Engl. J. Med..

[5] Esteva A., Kuprel B., Novoa R.A., Ko J., Swetter S.M., Blau H.M., Thrun S. (2017). Dermatologist-level classification of skin cancer with deep neural networks. Nature.

[6] Litjens G., Kooi T., Bejnordi B.E., Setio A.A.A., Ciompi F., Ghafoorian M., van der Laak J.A., van Ginneken B., Sánchez C.I. (2017). A survey on deep learning in medical image analysis. Med. Image Anal..

[7] Li L.Y., Isaksen A.A., Lebiecka-Johansen B., Funck K., Thambawita V., Byberg S., Andersen T.H., Norgaard O., Hulman A. (2024). Prediction of cardiovascular markers and diseases using retinal fundus images: A scoping review. Eur. Heart J. Digit. Health.

[8] Chikumba S., Hu Y., Luo J. (2023). Deep learning-based fundus image analysis for cardiometabolic and cardiovascular disease prediction: A review. Ther. Adv. Chronic Dis..

[9] Silberberg S., Dip O.D. (2026). The Oculomics paradigm: A comprehensive review. Optom. Times J..

[10] Gulshan V., Peng L., Coram M., Stumpe M.C., Wu D., Narayanaswamy A., Venugopalan S., Widner K., Madams T., Cuadros J. (2016). Development and Validation of a Deep Learning Algorithm for Detection of Diabetic Retinopathy in Retinal Fundus Photographs. JAMA.

[11] Zhou Z., Wang J. (2025). A Narrative Review of Multimodal Data Fusion Strategies for Precision Risk Prediction in Coronary Artery Disease: Advances, Challenges, and Future Informatics Directions. Rambam Maimonides Med. J..

[12] Topol E.J. (2019). High-performance medicine: The convergence of human and artificial intelligence. Nat. Med..

[13] Ramesh A.N., Kambhampati C., Monson J.R., Drew P.J. (2004). Artificial intelligence in medicine. Ann. R. Coll. Surg. Engl..

[14] Mhamdi L., Dammak O., Cottin F., Ben Dhaou I. (2022). Artificial Intelligence for Cardiac Diseases Diagnosis and Prediction Using ECG Images on Embedded Systems. Biomedicines.

[15] Al-Absi H.R.H., Islam M.T., Refaee M.A., Chowdhury M.E.H., Alam T. (2022). Cardiovascular Disease Diagnosis from DXA Scan and Retinal Images Using Deep Learning. Sensors.

[16] Fatima M., Pachauri P., Akram W., Parvez M., Ahmad S., Yahya Z. (2024). Enhancing retinal disease diagnosis through AI: Evaluating performance, ethical considerations, and clinical implementation. Inform. Health.

[17] Zhao R., Gillani S. (2026). AI in ophthalmology: A bibliometric analysis of retinal imaging innovations and global research collaboration. Photodiagnosis Photodyn. Ther..

[18] Haruna Y., Qin S., Hamman Adama Chukkol A., Abdu Yusuf A., Bello I., Lawan A. (2025). Exploring the synergies of hybrid convolutional neural network and Vision Transformer architectures for computer vision: A survey. Eng. Appl. Artif. Intell..

[19] Gencer G., Gencer K. (2025). Advanced retinal disease detection from OCT images using a hybrid squeeze and excitation enhanced model. PLoS ONE.

[20] Chen X., Yang C., Mo J., Sun Y., Karmouni H., Jiang Y., Zheng Z. (2024). CSPNeXt: A new efficient token hybrid backbone. Eng. Appl. Artif. Intell..

[21] Muthuraja M., Shanthi N., Aravindhraj N., Indhuja T., Madhuvarshini M., Hariharan J. (2026). A Vessel-Aware Deep Learning Framework for Cardiovascular Risk Prediction using Retinal Images. Proceedings of the Fourth International Conference on Augmented Intelligence and Sustainable Systems (ICAISS).

[22] Liu Z., Sunar M.S., Tan T.S., Hitam W.H. (2025). Deep learning for retinal vessel segmentation: A systematic review of techniques and applications. Med. Biol. Eng. Comput..

[23] Celik C., Yücedağ İ. (2026). The role of deep learning-based algorithms in retinal image processing: A review. Multimed. Tools Appl..

[24] Sun X., Guan S., Wu L., Zhang T., Ying L. (2025). A multimodal deep learning framework for automated major adverse cardiovascular events prediction in patients with end-stage renal disease integrating clinical and cardiac MRI data. Displays.

[25] Thirumalai M.C., Srivastava G. (2019). Effective Heart Disease Prediction Using Hybrid Machine Learning Techniques. IEEE Access.

[26] Sharean T.M.A.M., Johncy G. (2022). Deep learning models on Heart Disease Estimation—A review. J. Artif. Intell..

[27] Tiwari A., Chugh A., Sharma A. (2022). Ensemble framework for cardiovascular disease prediction. Comput. Biol. Med..

[28] Deng M., Wang C., Tang M., Zheng T. (2018). Extracting cardiac dynamics within ECG signal for human identification and cardiovascular diseases classification. Neural Netw..

[29] Elsayed N., Osman Y.B.M., Li C., Liu J., Si W., Zhang J., Wang S. (2025). Automating vessel segmentation in the heart and brain: A trend to develop multi-modality and label-efficient deep learning techniques. Biomed. Signal Process. Control.

[30] Aburass S., Dorgham O., Al Shaqsi J., Abu Rumman M., Al-Kadi O. (2025). Vision Transformers in Medical Imaging: A Comprehensive Review of Advancements and Applications Across Multiple Diseases. J. Imaging Inform. Med..

[31] Nikhil S., Anjali T. Enhancing Cardiovascular Risk Prediction with Vision Transformer and Retinal Image Analysis. Proceedings of the 2024 3rd International Conference on Automation, Computing and Renewable Systems (ICACRS).

[32] Abini M.A., Priya S.S.S. (2026). DRD-ViT: A dual-scale hybrid vision transformer with convolutional embedding for robust diabetic retinopathy detection in fundus images. Biomed. Signal Process. Control.

[33] Bi Q., Sun X., Yu S., Ma K., Bian C., Ning M., He N., Huang Y., Li Y., Liu H. (2023). MIL-ViT: A multiple instance vision transformer for fundus image classification. J. Vis. Commun. Image Represent..

[34] Singh D., Agarwal S., Mishra S., Jain S., Bhargava B.K., Kalra D., Groppe S. (2025). Retinal Fundus Multi-Disease Image Classification Using Hybrid CNN-Transformer-Ensemble Architectures. Proceedings of the International Health Informatics Conference. IHIC 2023. Lecture Notes in Networks and Systems.

[35] Kaur H. (2025). A Hybrid CNN-Transformer Model for Enhanced Heart Disease Prediction. Proceedings of the 7th International Conference on Signal Processing, Computing and Control (ISPCC).

[36] Cheng Z., Wu Y., Li Y., Cai L., Ihnaini B. (2025). A Comprehensive Review of Explainable Artificial Intelligence (XAI) in Computer Vision. Sensors.

[37] Fraz M.M., Remagnino P., Hoppe A., Uyyanonvara B., Rudnicka A.R., Owen C.G., Barman S.A. (2012). An ensemble classification-based approach applied to retinal blood vessel segmentation. IEEE Trans. Biomed. Eng..

[38] Bhardwaj R., Sethi A., Nambiar R. (2014). Big data in genomics: An overview. Proceedings of the 2014 IEEE International Conference on Big Data (Big Data), Beijing, China, 4–7 August 2014.

[39] Deepika K., Seema S. (2016). Predictive analytics to prevent and control chronic diseases. Proceedings of the 2016 2nd International Conference on Applied and Theoretical Computing and Communication Technology (iCATccT), Bangalore, India, 21–23 July 2016.

[40] Islam M.S., Jahan I. Diabetic Retinopathy and Retinal Diseases Fundus Image Dataset. Mendeley Data 2025, V1. https://data.mendeley.com/datasets/6ry9n79gb4/1.

[41] Karthik M., Dane S. APTOS 2019 Blindness Detection. https://www.kaggle.com/competitions/aptos2019-blindness-detection/data.

